# Converter Capacitor Temperature Estimation Based on Continued Training LSTM under Variable Load Conditions

**DOI:** 10.3390/s24134304

**Published:** 2024-07-02

**Authors:** Xiaoteng Dai, Yiqiang Chen, Jie Chen, Ruichang Qiu

**Affiliations:** 1 School of Electrical Engineering, Beijing Jiaotong University, Beijing 100044, China; 2 China Electronic Product Reliability and Environmental Testing Research Institute, Guangzhou 510610, China; yiqiang-chen@hotmail.com (Y.C.); jiechen@bjtu.edu.cn (J.C.); rchqiu@bjtu.edu.cn (R.Q.)

**Keywords:** capacitor, temperature estimation, LSTM algorithm, continued training

## Abstract

Capacitors are crucial components in power electronic converters, responsible for harmonic elimination, energy buffering, and voltage stabilization. However, they are also the most susceptible to damage due to their operational environment. Accurate temperature estimation of capacitors is essential for monitoring their condition and ensuring the reliability of the converter system. This paper presents a novel method for estimating the core temperature of capacitors using a long short-term memory (LSTM) algorithm. The approach incorporates a continued training mechanism to adapt to variable load conditions in converters. Experimental results demonstrate the proposed method’s high accuracy and robustness, making it suitable for real-time capacitor temperature monitoring in practical applications.

## 1. Introduction

Power electronic converters serve as interfaces between power sources (such as the grid, wind turbines, and solar photovoltaics) and loads (such as transmission systems, motor drives, electric vehicles, and residences). As power electronic converters are increasingly employed in power systems for interfacing purposes, it is imperative to maintain their reliability to ensure system stability. Among the components used in these power converters, capacitors exhibit the highest degradation and breakdown rates due to wear-out failures and short lifespans [1]. Among all types of capacitors, aluminum electrolytic capacitors (AEC) have advantages such as high energy density and low cost, which are widely used in the DC-link of converters, playing a role in voltage support, harmonic absorption, and energy buffering. The electrical characteristics (capacitance and ESR), as well as the lifespan of AECs, are closely related to temperature. As the temperature increases due to thermal expansion and contraction, the effective contact area between the electrolyte and the electrodes increases, leading to a rise in the effective capacitance. Additionally, with the temperature rise, the thermal motion of ions in the electrolyte accelerates, which increases conductivity and subsequently reduces the equivalent series resistance (ESR). However, the temperature increase also accelerates the evaporation rate of the electrolyte, thereby speeding up the aging process of the electrolytic capacitor. According to the empirical formula derived from the Arrhenius equation, for every 10 °C increase in temperature, the lifespan of the electrolytic capacitor is halved. This makes the temperature assessment of capacitors one of the research hotspots in the field of capacitor condition monitoring.

Over the past few decades, scholars have proposed their own approaches for capacitor temperature assessment. Thermodynamic modeling is one of the common methods used in this regard. A thermodynamic model based on the Foster model has been established in [2]. Model parameters are obtained through experimental measurements. The heat dissipation power is calculated using the ESR determined by a table lookup method, and the capacitor current is measured with a current sensor. Using a B–H analyzer, the variation characteristics of the capacitor’s ESR value with temperature and current are measured in [3]. Then, with a querying method, the thermal resistance of the capacitor is calculated to establish a thermodynamic model. The current of the capacitor is analyzed at different frequencies to compute the power loss and core temperature of the capacitor. The core temperature of the capacitor is estimated through thermodynamic modeling and heat dissipation power calculation in [4]. The heat dissipation power is calculated based on measured ESR characteristics and current analysis. A thermodynamic model for a capacitor group was established based on the Foster network model, with a focus on the thermodynamic interactions between capacitors [5]. Model parameters were confirmed through finite element simulation and experimental testing. A thermal network model was adopted to investigate the thermal coupling between the DC bus and capacitors [6]. Parameters were determined using optimization algorithms and data training. Simulation modeling methods have advantages in assessing the steady-state temperature and its distribution. AECs were subjected to finite element simulation using ANSYS 15.0, investigating the temperature distribution of the capacitors under different ripple currents in steady-state conditions [7]. The finite element modeling of metalized film capacitors was simplified for calculating the lumped-parameter thermal model of the capacitor [8]. The extracted Foster or Koel network is combined with the electrical model to evaluate the temperature of the capacitors in circuit simulators such as SPICE or Simulink. Finite element simulation was used to assess the temperature distribution under steady-state conditions for high-voltage parallel film capacitors [9]. A novel finite element simulation modeling method for large capacitors was proposed in [10], taking into account the internal geometry and anisotropic material properties of the capacitors. The method of combined simulation, utilizing both finite element simulation and Matlab simulation in conjunction, iterates on the model’s ESR degradation, power loss, and temperature through data updates, achieving more precise capacitor temperature simulation over long time scales [11]. The temperature characteristics of capacitors are also commonly used for temperature estimation. By measuring the temperature characteristic curve of capacitors and monitoring their capacitance, the core temperature of capacitors can be obtained using a table lookup method [12,13,14]. Some researchers have also developed novel high-precision temperature sensors for measuring the temperature distribution of high-voltage film capacitors [15]. In addition, neural networks are increasingly being widely applied. Four different neural network approaches were utilized to estimate the environmental temperature of capacitors in an air-cooled power supply, validating the applicability of neural networks [16].

Although the aforementioned studies have achieved certain results, they also have evident issues. The construction of models and the determination of parameters are critical factors influencing the accuracy of thermodynamic models. The method of model construction can also affect thermodynamic simulation. A detailed modeling approach may enhance simulation accuracy, but it could also impact computational efficiency. Furthermore, most methods focus on simulating capacitor temperatures under steady-state conditions. Methods based on capacitor temperature characteristics are affected by capacitor aging, necessitating updates to characteristic curves during usage. Errors in capacitor monitoring directly affect temperature monitoring accuracy. Although sensor-based methods can directly measure internal capacitor temperatures, they require embedding sensors during the manufacturing stage. Neural network-based methods can avoid complex modeling, but current research has not addressed capacitor temperature variations under complex load changes.

This paper proposed a capacitor hotspot temperature estimation scheme based on the long short-term memory (LSTM) algorithm. This scheme enables accurate assessment of capacitor core temperature under conditions of frequent load changes. The method offers the following advantages:

(a) It does not require understanding the internal structure of the capacitor, as it is based on data training, thus avoiding the complexity of modeling.
(b) It is more closely aligned with the actual operating conditions of the system, especially under multiple types of load changes.
(c) Through the continued training mechanism, the proposed method can quickly and effectively learn capacitor temperature rise data with new characteristics. This approach enhances the model’s output accuracy and significantly reduces training time.

The section arrangement of this paper is as follows: Section 2 introduces the structure of the LSTM model and continued training mechanism. Section 3 details the temperature rise experiment for the capacitor and the implementation of the continued training mechanism. Section 4 presents the validation results and discussion. Finally, Section 5 concludes the paper.

## 2. LSTM Deep Neural Network

### 2.1. Structure of LSTM

**Figure 1:**
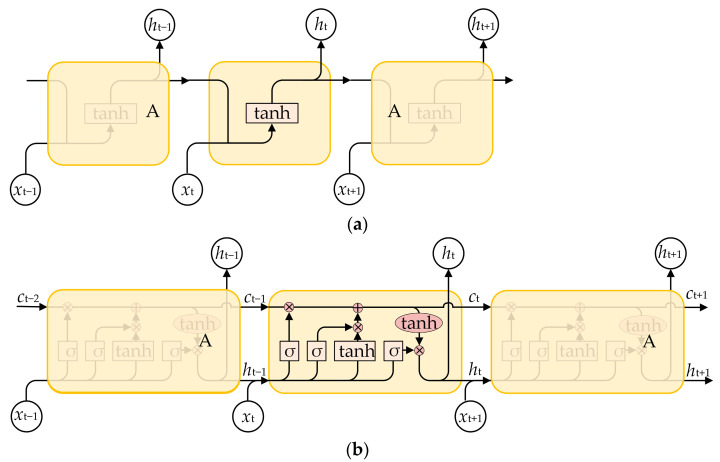
Structures of RNNs. (**a**) Vanilla RNN. (**b**) LSTM.

A recurrent neural network (RNN) is a type of recursive neural network that takes sequence data as input and recursively processes it in the direction of sequence evolution, with all nodes (recurrent units) connected in a chain-like manner [17]. RNNs are widely used for handling time-series tasks, such as natural language processing [18], stock prediction [19], and power demand analysis [20,21]. The structures of RNNs are illustrated in Figure 1. It can be seen that the output *h*_t_ not only depends on the current input *x*_t_ but also on *h*_t−1_ from the previous time step, which can be expressed as follows:

(1) $$h_{t}=\sigma \left(x_{t}\times \omega_{xt}+h_{t-1}\times \omega_{ht}+b\right)$$

where ω*_xt_* and ω*_ht_* are the weights for *x_t_* and *h_t_*_._ This endows RNNs with short-term memory capabilities, making them perform well for time-series problems.

However, traditional RNNs may encounter the vanishing or exploding gradient problem when dealing with a long time series, making it difficult for them to capture long-term dependencies [22]. To address this issue, the LSTM algorithm was proposed by Hochreiter and Schmidhuber [23]. The structure of the LSTM algorithm, after further development, is shown in Figure 1b. The LSTM algorithm introduces three gates: the forget gate *f*_t_, input gate *i*_t_, and output gate *o*_t_. These gates are used to control the flow of information and memory updates. The forget gate controls whether information from the previous time step’s memory state should be retained. The input gate determines how new input information should be updated into the memory state. The output gate determines how information from the memory state should be passed to the next time step or output layer. In addition, the LSTM algorithm includes a cell state responsible for transmitting information throughout the network and storing long-term memories. The cell state allows the network to retain and update information more effectively without being affected by the vanishing or exploding gradient problem. They can be expressed as follows:

(2) $$i_{t}=\sigma (W_{i}\cdot \left[h_{t-1},x_{t}\right]+b_{i})$$

(3) $$o_{t}=\sigma (W_{o}\cdot \left[h_{t-1},x_{t}\right]+b_{o})$$

(4) $$f_{t}=\sigma (W_{f}\cdot \left[h_{t-1},x_{t}\right]+b_{f})$$

(5) $$C_{t}=f_{t}\times C_{t-1}+i_{t}\times \tilde{C}$$

(6) $$\tilde{C}=\tanh\left(W_{C}\cdot \left[h_{t-1},x_{t}\right]+b_{C}\right)$$

(7) $$h_{t}=o_{t}\cdot \tanh\left(C_{t}\right)$$

The LSTM model transmits linear and recurrent information through the internal cell state (*C*_t_) and outputs nonlinearly to the output state (*h*_t_) of the hidden layer. The computation of the LSTM algorithm’s unit structure is as follows. First, the previous time step’s output state (*h*_t−1_) and the combined input gate (*x*_t_) are used to calculate the three gates and the candidate state. Second, the forget gate (*f*_t_) and the input gate (*i*_t_) are combined to calculate the memory cell (*C*_t_). Finally, the output gate (*o*_t_) transmits information about the internal state to the output state (*h*_t_). Compared with traditional RNN, which has only one hidden state at each time step, the LSTM algorithm introduces a cell state and three gate control states, enabling it to better handle long sequence information [24].

**Figure 2:**
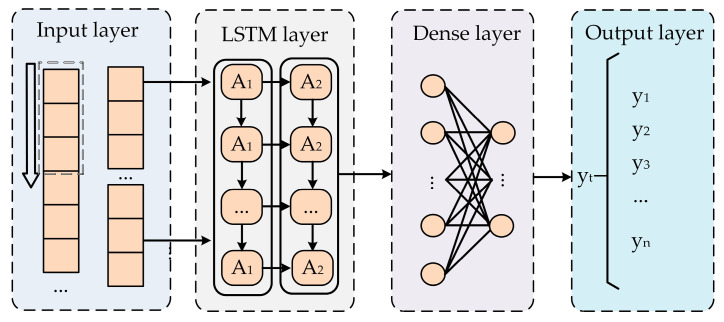
The LSTM algorithm’s temperature estimation model architecture.

The proposed model structure is illustrated in Figure 2. The model comprises an input layer, two hidden LSTM layers, two dense layers with linear activation functions, and an output layer. The input layer normalizes the collected data and partitions it into training, validation, and test sets. The LSTM algorithm layers process the partitioned datasets, iteratively updating parameters through training. The dense layers connect each neuron from the input layer to every neuron in the output layer, enabling a full connection between the input and output layers. This connection method allows the model to better learn the relationship between inputs and outputs, thereby enhancing the model’s generalization capability.

Based on this structure, the input variables for the model are DC current, ambient temperature (AT), and shell temperature (ST), while the output variable is the capacitor hotspot temperature (HST).

### 2.2. Dataset Creation and Partitioning

**Figure 3:**
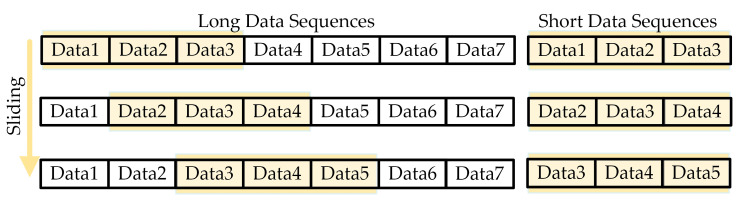
Mechanism of the sliding window method.

After the data sampling, the data becomes a continuous multidimensional time-series signal. The next step is to create and partition the dataset using the sliding window method. The sliding window method is a commonly used signal processing technique for time-series data or time-series signals. Its working principle is illustrated in Figure 3. The main idea is to define a fixed-size window marked as bold color in figure and slide it over the time-series signal to extract features within the window. The steps of the sliding window method are as follows:

With the sliding window method, we can capture features of different time periods in multidimensional time-series signals and construct and expand the limited dataset to provide richer input information for subsequent analysis, modeling, and prediction.

**Figure 4:**
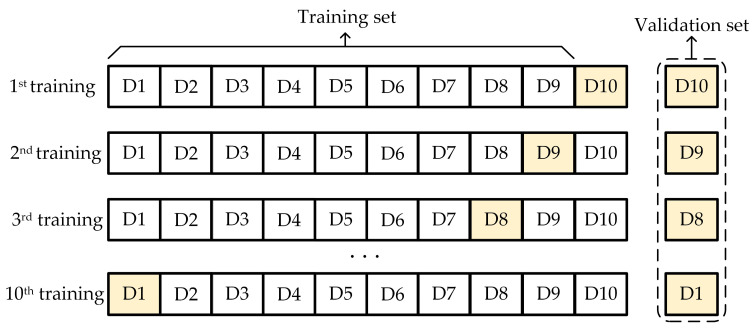
Mechanism of the cross-validation.

The generated short-sequence data are divided into training, validation, and testing sets according to certain proportions. The order of sample data within the dataset is randomly shuffled, ensuring the temporal relationships within the short sequences while reducing the temporal characteristics between sequences. This allows the model to more comprehensively learn the patterns of the data itself, enhancing its generalization ability and avoiding overfitting. Additionally, to better evaluate model performance and improve its generalization ability, this study adopts a 10-fold cross-validation approach, as shown in Figure 4. The dataset is equally divided into 10 mutually exclusive subsets, with one subset selected as the testing set each time and the remaining subsets used as the training set. The model is trained and evaluated 10 times repeatedly, and the average of the performance metrics from these 10 evaluations is calculated to obtain the final performance indicator of the model.

It is worth emphasizing that, to ensure the objectivity of the test results, each sample is assigned to only one dataset, meaning the samples in the test dataset do not overlap with those in the training dataset. This partitioning approach helps evaluate the model’s performance on unseen data and improve its generalization ability.

### 2.3. Continued Training Mechanism

In the context of deep learning model training, the continued training mechanism allows for further optimization of model parameters based on an already trained model. This mechanism is particularly suitable for scenarios such as the following:

(a) Extended training times—Training a complete model may take days or even weeks, and it may not be feasible to complete the training in one go.
(b) Dynamic data updates—In practical applications, data may continually update or increase, necessitating further model optimization based on new data.
(c) Performance enhancement—Continued training can potentially improve the performance of the existing model.

The basic principle of continued training involves saving the current state of the model, including model parameters (weights and biases) and optimizer state (momentum and learning rate scheduler). Subsequent training resumes from this saved state rather than starting from scratch. This process includes the following steps:

(a) Saving the model state—At the end of the initial training, save the model parameters and optimizer state.
(b) Loading the model state—At the start of continued training, load the previously saved model parameters and optimizer state.
(c) Continued training—Continue the training process, updating the model parameters based on the loaded state.

By conducting continued training on different data, it is possible to avoid repetitive training from scratch, significantly saving time and computational resources. In practical engineering applications, data are often dynamically changing. The continued training mechanism allows the model to undergo incremental training when new data arrives, enabling the model to promptly adapt to the latest data distribution, thereby maintaining good performance.

## 3. Experiment and Model Training

### 3.1. Experimental Setup

**Figure 5:**
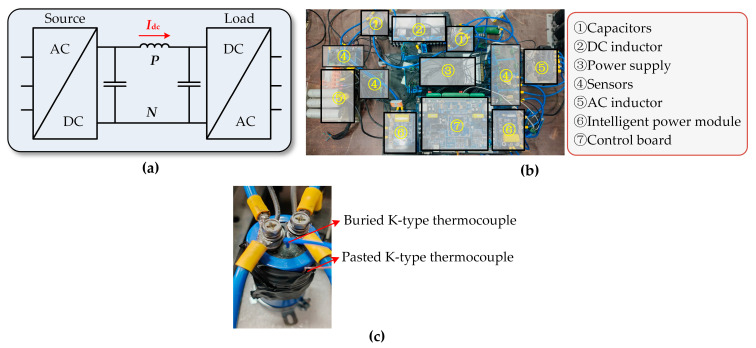
Schematic diagram of (**a**) circuit structure, (**b**) physical platform, and (**c**) capacitor under test with thermocouple.

In order to simulate the thermal stress of capacitors under complex load variations, an AC/DC/AC three-phase half-bridge converter test platform has been designed and implemented. Two converters adopt the same topology structure, as shown in Figure 5a,b. Two intelligent power modules (IPM) are respectively used to form the source converter and load. The whole system is controlled by a 32-bit DSP chip and a FPGA chip. The DSP chip is responsible for the main control functions of the system, including sampling, control, protection, computation, communication, etc. FPGA plays a role in improving the driving capability of the DSP chip, while also being responsible for hardware protection by fast blocking of driving signals. Sensors are used to measure the voltage and current of AC and DC for converter control. Load side control adopts current loop control to simulate load power. The source side control adopts the voltage outer loop, and current inner loop double closed loop control to simulate the regulation of the system to the DC network voltage. Sensors are used to measure the voltage and current of AC and DC to control the inverters. The corresponding data of the system are uploaded to the upper computer through the SCI serial communication module of the control board. The specific details of the platform are shown in Table 1.

The capacitors used are from Nichicon, LNR2C222MSE, with a rated voltage of 160 V, rated capacitance of 2200 μF, tangent of loss angle of 0.15, and leakage current of 1.77 mA. The training of the model requires the core temperature and shell temperature of the capacitors. To obtain the core temperature of the capacitors, a K-type thermocouple is embedded in the center of the capacitors during the manufacturing process, as shown in Figure 5c. The shell temperature is obtained by externally attaching a K-type thermocouple. Data are collected and uploaded to the host computer through a data acquisition unit 34970A from AGILENT.

### 3.2. Experiment Schedule

The experimental testing is divided into four parts: the constant load (CL), mission profile load (MPL), 10 s cycle random load (10sRL), and 5 s cycle random load (5sRL). Table 2 provides an overview of the data from the four types of experiments. The sampling frequency is 1 s, and there are a total of 59 sets of experimental data, with an approximate total experiment duration of 108 h. The collected data include capacitor hotspot temperature, shell temperature, ambient temperature, and DC current. In this subsection, we provide a detailed presentation of some of the experimental data obtained from the various test scenarios.

**Figure 6:**
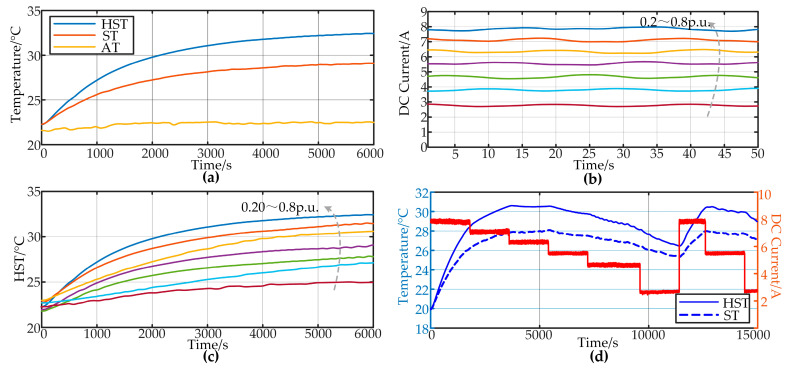
Experiment data of (**a**) single group of 0.8 p.u. load, (**b**) DC current, (**c**) CL data, and (**d**) MPL data.

The first part involves temperature rise experiments under constant power conditions, simulating fixed loads such as ventilation and lighting. The capacitors undergo temperature rise experiments under different load depths. Constant loads are divided into seven groups, ranging from load depths of 0.2 to 0.8. One set of data in the data groups is shown in Figure 6a–c, including capacitor temperature data and DC current waveform. It can be observed that the rate of increase in HST and the final temperature both increase with the increase in load depth. This is because with a larger load, there is a greater amount of harmonic current contained in the output current of the converter. The capacitor endures more harmonic stress, resulting in more heat generation.

The second part involves long-term temperature rise experiments under variable load conditions, simulating applications such as rail transportation. The capacitors undergo temperature rise testing over a long time scale at various power levels set for multiple mission profiles. The DC current and HST data for one group of the data groups are shown in Figure 6d. It can be observed from the figure that there are significant changes in the HST with variations in the DC current at different time intervals. This indicates a strong coupling relationship between the capacitor HST and the DC current.

**Figure 7:**
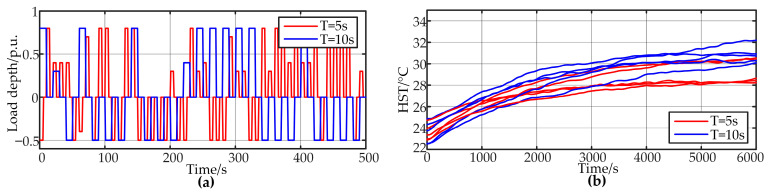
Random load experiment data (**a**) Random load depth (**b**) HST.

The third and fourth parts involve experiments under conditions of frequent and random power fluctuations, simulating high-frequency load variations such as those encountered in electric vehicles, resulting in more complex fluctuations in hotspot temperature. The load inverter randomly fluctuates within a predetermined power range at fixed intervals. The fluctuation range of the load power is from −0.5 to −0.3 and from 0.3 to 0.8. The fluctuation periods are divided into 5 s and 10 s, with each group consisting of twn experiments. Figure 7 provides temperature rise data for five sets of experiments for both 5 s and 10 s fluctuation periods, along with an example of random load depth on the load side. It can be observed that under these load conditions, the hotspot temperature exhibits more complex fluctuations, posing higher demands on the model.

### 3.3. Batches Training of Models Based on Continued Training Mechanism

**Figure 8:**
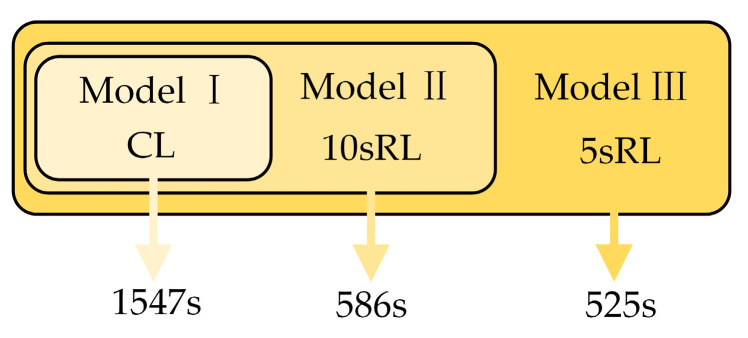
Model training process based on continued training.

The sampled data are utilized to train the established LSTM model for estimating the capacitor hotspot temperature. The model training process employs a continued training mechanism that is conducted in batches according to different load types, which is shown in Figure 8. Model I is trained using data from CL. Model II builds upon Model I by further training it with 10sRL data using the continued training mechanism. Similarly, Model III builds upon Model II by further training it with 5sRL data. The training time of Model I, Model II, and Model III; is 1547 s, 586 s, and 525 s, respectively, based on continued training. However, training Model II; and Model III; from scratch takes 1923 s and 2456 s, respectively. The training method based on the continued training mechanism saves 69.5% and 78.6% of the training time, respectively

By following this continued training approach, each model incrementally improves upon its predecessor, ensuring comprehensive training and robust performance across different load conditions.

## 4. Validation Results and Discussion

### 4.1. Validation of Model Ⅰ

**Figure 9:**
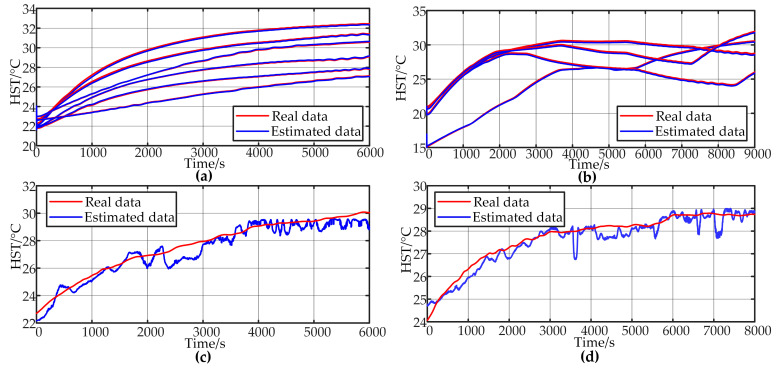
Model I test results. (**a**) CL validation. (**b**) MPL validation. (**c**) 10sRL validation. (**d**) 5sRL validation.

The CL data testing results for Model I are illustrated in Figure 9a. From the figures, it can be observed that the model’s output closely aligns with the actual values, demonstrating minimal error and accurately completing the temperature estimation task. The mean absolute error (MAE) and mean relative error (MRE) are 0.02293 and 0.0881%, respectively, indicating high accuracy.

Testing Model I with MPL data yields the results shown in Figure 9b. Despite MPL data not being included in the training process of Model I, the output maintains good accuracy. For the four data sets, the MAE and MRE are 0.06971 and 0.2457%, respectively, which are acceptable.

Model I was tested with data characterized by random and frequent load fluctuations; the results are shown in Figure 9c,d. Without being trained on random load data, Model I can only roughly estimate the temperature trend. The output exhibits significant fluctuations, with the evaluation error exceeding 1 °C in some periods. Additionally, influenced by high-frequency fluctuations in the DC current input, the output displays small ripple variations. The MAE for the 10 s random fluctuation test data is 0.4419, and the MRE is 1.6057%. For the 5 s random fluctuation test data, the MAE is 0.3497, and the MRE is 1.2442%, indicating that the temperature estimation accuracy for high-frequency fluctuating loads does not meet the required standards. Therefore, incremental training is necessary to adapt the model to new temperature data distribution patterns.

### 4.2. Validation of Model Ⅱ

**Figure 10:**
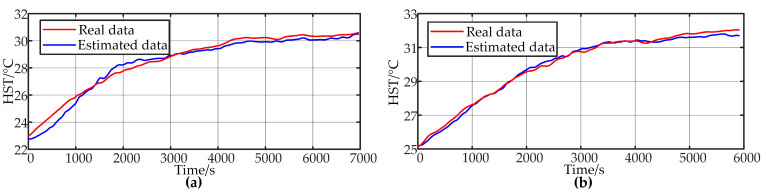
Model Ⅱ test results. (**a**) 10sRL validation. (**b**) 5sRL validation.

Model II was tested using the 10sRL data, and the results are shown in Figure 10a. The figure clearly shows that after incremental training, compared to Model I, the output more closely matches the actual values, with an MAE and MRE of 0.128 and 0.4287%, respectively, indicating a significant improvement in accuracy. Furthermore, the Model Ⅱ was tested with untrained 5sRL data, as shown in Figure 10b. Although Model II was trained with 10 s load random fluctuation data, it can still estimate the temperature with a certain degree of accuracy for higher frequency load changes. However, the accuracy decreases, with an MAE and MRE of 0.2952 and 1.0748%, respectively, which, while within an acceptable range, still leaves room for improvement.

### 4.3. Validation of Model Ⅲ

**Figure 11:**
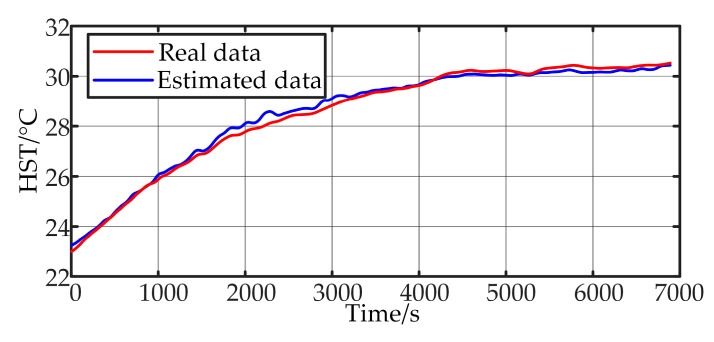
Model Ⅲ test results of 5sRL validation.

Model III was tested with 5sRL data, and the results are shown in Figure 11. Compared to Model II, Model III exhibits a significant improvement in accuracy. It is capable of accurately estimating the capacitor temperature even under high-frequency load variations. The MAE and MRE are 0.1521 and 0.5353%, respectively.

### 4.4. Validation under Different Temperature Ranges

**Figure 12:**
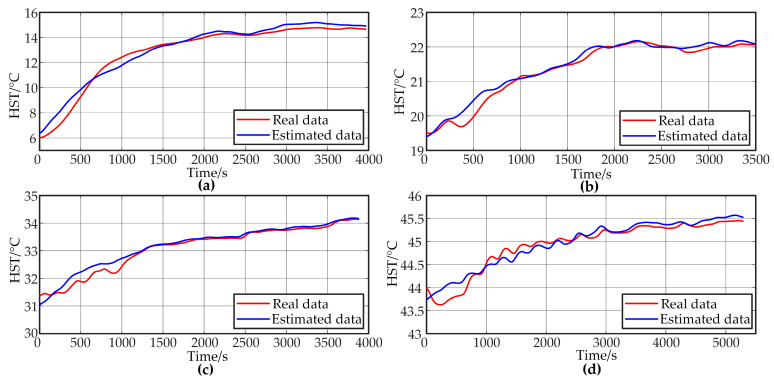
Validation of Model III in different temperature ranges: (**a**) 5 °C, (**b**) 15 °C, (**c**) 30 °C and (**d**) 40 °C.

The ambient temperature range for the above experiments was between 20 °C and 25 °C. In order to verify the performance of the model in different ambient temperature ranges, related experiments were conducted in this subsection. The capacitor was placed in a thermostat with ambient temperatures set at 5 °C, 15 °C, 30 °C 40 °C. The power of the converter was set to 5sRL. Experimental data were collected, and the capacitor core temperature was estimated using Model III. The results of the model evaluation are shown in Figure 12.

Figure 12 shows that the magnitude and rate of increase in the hotspot temperature decreases as the ambient temperature rises. At an ambient temperature of 5 °C, the hotspot temperature can increase by approximately 9 °C within 3000 s. However, at an ambient temperature of 40 °C, the hotspot temperature only rises by about 2 °C over 5000 s. The temperature rise curves exhibit distinct characteristics across different temperature ranges.

It is noteworthy that, despite the fact that the data within these temperature ranges were not included in the model training, the prediction results of the model maintained a high degree of accuracy. For ambient temperatures of 15 °C and 30 °C, which are close to the training data conditions, the evaluation accuracy remained high, with MAEs of 0.1001 and 0.1104, respectively. For the 40 °C test, due to the minimal temperature rise, the output accuracy did not significantly decline, yielding an MAE of 0.1069. However, for the 5 °C test, where the temperature rise magnitude and rate were markedly higher than the training data, the evaluation accuracy decreased, resulting in an MAE of 0.3568. With the continued training mechanism, the model can learn the characteristics of the data in this temperature range to reduce this error.

### 4.5. Discussion

From the above validation, it is evident that the proposed evaluation scheme effectively estimates the hot spot temperature of capacitors. However, different load types and temperature ranges result in varying characteristics of the capacitor’s hot spot temperature rise curves, which can affect the model’s accuracy. By employing the continued training mechanism used in this study, it is possible to supplement the model with new data features on the existing model, thereby saving training time and significantly enhancing the model’s accuracy. The final trained model achieves high-precision evaluations with a MAE of approximately 0.1 in the temperature range of 15 °C to 45 °C, even under complex load variations, demonstrating substantial practical engineering significance.

## 5. Conclusions

This paper proposes an LSTM-based method for estimating the hot spot temperature of capacitors, using DC current, shell temperature, and ambient temperature as input data. This paper addresses the challenges of determining thermal parameters and the complexity of traditional thermodynamic models and introduces an LSTM temperature estimation model training process. With the established experimental platform, long-term fixed load experiments, mission profile load experiments, and high-frequency random load variation experiments were conducted to simulate the electrothermal stress that capacitors may encounter in different application scenarios. The collected data were used for model training. 

In terms of model training, a continued training mechanism was employed, adopting progressive training under different operating conditions to incrementally train the existing models. This approach improved the robustness and accuracy of the model when dealing with data with varying characteristics, significantly reducing the time required for model updates. The trained model was used to evaluate the hot spot temperature of capacitors under various load conditions and different ambient temperature ranges, demonstrating high evaluation accuracy. The final model achieved high-precision evaluations with a MAE of approximately 0.1 under complex varying load conditions. For fixed loads and mission profile loads, the model demonstrated even higher evaluation accuracy.

## Figures and Tables

**Table 1:** The experiment platform setup.

Experimental Parameters	Setup
AC voltage	50 V_rms_
DC voltage	110 V
DC filter inductor	3.2 mH
AC filter inductor	4.2 mH
Intelligent Power Module (IPM)	7MBP50RA120-55
Digital Signal Processing (DSP)	TMS320F28377D
Field Programmable Gate Array (FPGA)	10CL080YF484I7G
Analog-to-Digital Converter (ADC)	16-bit AD7656
Switching frequency	1.25 kHz

**Table 2:** Overview of load experiment sample data.

Load Types	Data Groups	Total Sample Time
Constant load (CL)	35 groups	210,687 s
Mission profile load (MPL)	4 groups	44,912 s
10 s cycle random load (10sRL)	10 groups	68,423 s
5 s cycle random load (5sRL)	10 groups	64,425 s

## Data Availability

Dataset available on request from the authors.
